# Vertical Distribution of Ozone and Nitrogenous Pollutants in an Air Quality Class I Area, the San Gorgonio Wilderness, Southern California

**DOI:** 10.1100/tsw.2002.73

**Published:** 2002-01-05

**Authors:** Rocio Alonso, Andrzej Bytnerowicz, Michael Arbaugh

**Affiliations:** USDA Forest Service, Pacific Southwest Research Station, 4955 Canyon Crest Drive, Riverside, CA 92507, USA

**Keywords:** ozone, ammonia, nitric acid, nitrogen oxides, elevation gradient, passive samplers, air pollution, monitoring, nitrogen deposition

## Abstract

Information about spatial and temporal distribution of air pollutants is essential for better understanding of environmental stresses affecting forests and estimation of potential risks associated with air pollutants. Ozone and nitrogenous air pollutants were monitored along an elevation gradient in the Class I San Gorgonio Wilderness area (San Bernardino Mountains, California, U.S.) during the summer of 2000 (mid-June to mid-October). Passive samplers were exposed for 2-week periods at six sampling sites located at 300 m intervals ranging from 1200 to 2700 m elevation. Elevated concentrations of ozone were found in this area with summer 24-h hourly means ranging from 53 to 59 ppb. The highest ozone concentrations were detected in the period July 25 to August 8, reaching values of 64 to 72 ppb expressed as 2-week mean. Passive-sampler ozone data did not show a clear relationship with elevation, although during the periods with higher ozone levels, ozone concentrations were higher at those sites below 2000 m than at sites located above that elevation. All nitrogenous pollutants studied showed a consistent decrease of concentrations with elevation. Nitrogen dioxide (NO_2_) levels were low, decreasing with increasing elevation from 6.4 to 1.5 ppb summer means. Nitric oxide (NO) concentrations were around 1 to 2 ppb, which is within the range of the detection levels of the devices used. Nitric acid (HNO_3_) vapor concentrations were lower at higher elevations (summer means 1.9 to 2.5 μg m) than at lower elevations (summer means 4.3 to 5.1 μg m). Summer concentrations of ammonia (NH_3_) were slightly higher than nitric acid ranging from 6 μg m at the lowest site to 2.5 μg m registered at the highest elevation. Since complex interactions between ozone and nitrogenous air pollutants have already been described for forests, simultaneous information about the distribution of these pollutants is needed. This is particularly important in mountain terrain where no reliable models of air pollutant distribution exist.

## INTRODUCTION

Air pollution effects on forest ecosystems are considered an increasingly important environmental issue all over the world. The perceived health of forests has started to play an important role in policy decisions and strategies to control air quality that might have significant economic and ecological implications. The occurrence of greenhouse gas pollutants (carbon dioxide, ozone, nitrogen oxides and methane) continues to increase globally in phase with growing human population[1]. In 1977 the U.S. Congress implemented amendments to the Clean Air Act that established the goal of remedying existing visibility impairment, and preventing future impairment, from man-made pollution at 158 national parks and wilderness areas across the U.S. One of the potentially most impacted of these areas is the San Gorgonio Wilderness. This Class I wilderness area is located directly downwind of the Los Angeles-Orange County metropolitan area in the San Bernardino Mountains. Impacts of atmospheric pollution on forest tree populations have included increased mortality of sensitive genotypes leading to changes in population sensitivity, changes in the ability of a given species to compete with other species, changes in gene frequency and/or occurrence of rare alleles, and in the most extreme, localized population extinction[2].

The best-documented description of harmful effects of photochemical pollution on a forest ecosystem pertains to the San Bernardino Mountains in southern California. Since the 1950s, ozone ($O_3$) has been identified as the cause of chlorotic mottle symptoms on needles of sensitive species[3,4]. In 1978 the South Coast Air Quality Management District began to operate an ozone monitoring station at the western end of the San Bernardino Mountains. This station continues to operate at present and has the distinction of recording the highest ozone concentrations of any station in California[5]. Trees from heavily polluted areas exhibited reductions in radial growth and higher vulnerability to mortality from insect/disease stresses[6]. More recently, the effects of chronic atmospheric nitrogen (N) deposition on this area have also been documented[7]. Chronic deposition of N compounds to forest ecosystems may cause significant disturbances, such as changes in species composition, soil acidification, changes in soil fertility and nutrient imbalances and contamination of soils and water[8,9]. It has been postulated that elevated atmospheric N inputs may be a factor in mitigating $O_3$-induced reductions in tree growth[10]. However, long-term exposure of trees to oxidant pollution and nitrogen deposition is associated with high foliar injury and lower foliar retention accompanied by lower root biomass[11,12]. The decrease in root biomass in highly polluted areas may pose a long-term threat to forest sustainability[10].

Atmospheric nitrogen of anthropogenic origins consists of oxidized N compounds (mainly NO, $NO_2$, $HNO_3$, $NO_3^-$) from fossil fuel combustion by automobile traffic and industrial processes and of reduced N compounds ($NH_3$ and $NH_4^+$) mainly emitted from intensive agriculture. In the arid environment of southern California, dry deposition processes, as opposed to wet deposition during rainfall, provides as much as 90 to 95% of total nitrogen deposition to terrestrial ecosystems[13]. Dry N deposition in the highly urbanized area of Los Angeles Basin is dominated by oxidized N species.

Information about spatial and temporal distribution of air pollutants is essential for a better understanding of environmental stresses affecting forests and the estimation of potential risks associated with air pollutants. Distribution of air pollutants depends on pollutant species, atmospheric chemistry, landform and prevailing meteorological conditions. Models of air pollution distribution based on emission inventories and weather data have been developed for homogeneous continental areas[14]. However, these general pollution transport models cannot be applied in complex mountainous terrain where other types of models need to be developed.

Monitoring air pollution in remote locations has increased an interest in passive sampling techniques which do not require electric power to operate and are inexpensive and easy to use. Passive monitors integrate exposure over relatively long periods of time (days, weeks) to give cumulative exposure values. Several types of passive sampling systems have been developed for collecting different air pollutants. In general, they depend on diffusion of the measured pollutant to a collecting or indication medium[15]. The main disadvantages of this technique are that short-term fluctuations in air pollutant concentrations are not evident and that a possibility of interference from other atmospheric compounds exists.

Horizontal variability in the distribution of ozone and atmospheric nitrogen compounds has been described in the San Bernardino Mountains. A steep air pollution gradient spanning 55 km has been identified, with ozone and N deposition decreasing from west to east[16,17]. The incremental reduction in N deposition is more pronounced than decreases in ambient ozone due to the higher deposition velocities of $HNO_3$ and $NH_3$ compared to $O_3$[18]. However, little information exists on the atmospheric concentrations of pollutants in the San Gorgonio Wilderness area, especially the vertical distribution of these pollutants, i.e., changes in pollutant concentrations with increasing elevation, particularly for nitrogen compounds. A good understanding of distribution of air pollutants is essential to identify areas where vegetation may be at risk and for planning future management and preservation policies for these mountains.

The main goal of this study was to quantify the distribution of ozone and nitrogen compounds during summer months along an elevation gradient in the San Bernardino Mountains using a network of passive samplers.

## MATERIAL AND METHODS

The San Bernardino Mountains are situated approximately 90 km east from Los Angeles in southern California (Fig. 1). A well-established ambient pollution gradient exists with decreasing ozone and nitrogen concentrations from southwest to the northeast. In this study air pollution monitoring sites were established along the western slope of San Bernardino peak in San Gorgonio Wilderness area. This area is believed to be a moderately polluted area of the San Bernardino Mountains pollution gradient. Six sampling sites were established at 300 m intervals ranging from 1200 to 2700 m elevation (1223, 1502, 1826, 2111, 2428, and 2667 m). Sites were chosen in open-air areas where passive samplers could intercept optimal airflow. The samplers were located at about 2 m above ground level. This transect was sampled with passive samplers for periods of 2 weeks between June 15 and October 15, 2000. An additional plot at Barton Flats (2000 m elevation), located near the elevation gradient but on the north slope of the mountain, was also sampled during the same period in order to test ozone sampler accuracy by comparing results from passive samplers and from an electronic monitor.

Average ambient concentrations of $O_3$, $NO_2$, and $NO_x$ were measured with Ogawa passive samplers (Ogawa & Co., USA, Inc). Each sampler contained two replicate coated filters. Nitrite saturated filters were used to measure ozone exposure[19]. In the presence of ozone, nitrite is oxidized to nitrate that is extracted with nanopure water and determined with ion chromatography (Dionex 4000i analyzer). Ozone concentrations were calculated based on a calibration performed in the Sequoia National Park in 1999[20]. Commercially available filters were used for $NO_2$ (tri-

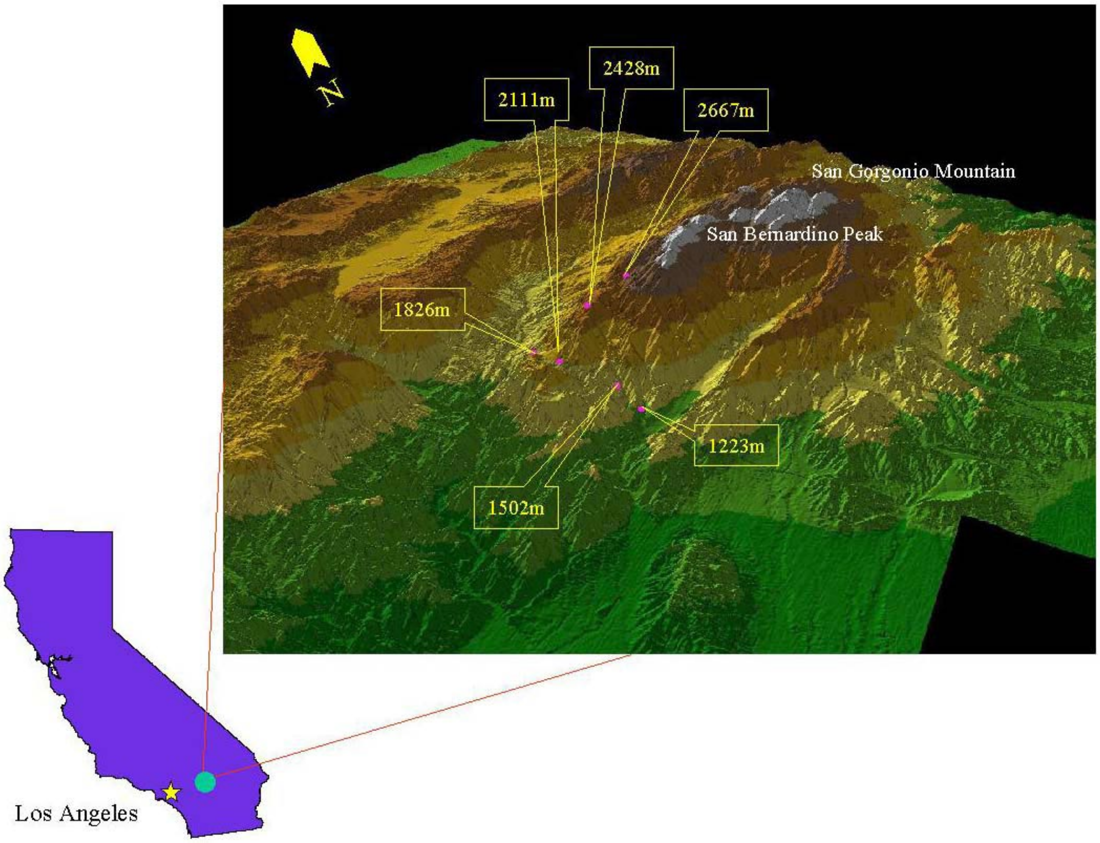

**FIGURE 1.** Location of the monitoring sites along an elevation gradient in the San Bernardino Mountains

(triethanolamine, TEA) and $NO_x$ (TEA and 2-phenyl-4,4,5,5-tetramethylimidazoline-3-oxide-1-oxyl, PTIO) determinations (Ogawa & Co., USA, Inc). Nitrite was extracted with nanopure water and analyzed colorimetrically with a Technicon TRAACS Autoanalyzer (Bran+Luebbe GmbH). Average concentrations were calculated following manufacturer instructions[21].

Similarly, 2-week-long average concentrations of $HNO_3$ vapor were monitored with passive samplers developed by Riverside Pacific Southwest Research Station USDA Forest Service. Four Nylasorb nylon filters (Pall Corporation, Gelman Lab, Michigan, U.S.) were hung under polyethylene caps protecting them from wind and rain and extracted with nanopure water. Nitric acid collected as nitrate was then determined by ion chromatography (Dionex 4000i analyzer). Samplers were calibrated against a honeycomb denuder system showing very high affinity to $HNO_3$ vapor ($R^2 = 0.99$[22]).

Ammonia were determined with diffusion tubes (Gradko International Ltd, U.K.) using 1% w/v sulphuric acid as collection reagent. Ammonia in the air is converted to ammonium ions on collecting pads of a sampler. Ammonium is extracted with nanopure water and analyzed by colorimetry (Technicon TRAACS Autoanalyzer). Four replicated diffusion tubes were used per site. Cumulative $NH_3$ dose was calculated using results of a calibration curve made against a honeycomb denuder system[23] ($R^2 = 0.989$, Fig. 2). The calibration was performed by exposing diffusion tubes to ambient $NH_3$ concentrations ranging from 13 to 35 µg $m^{-3}$ 24-h averages for a duration of 2 to 14 days. Average $NH_3$ concentrations were calculated by dividing the cumulative dose by time of exposure. When cumulative amount of ammonium collected on the diffusion tubes was above 0.45 µg per tube, ammonia concentrations obtained with tubes and honeycomb denuders showed an agreement within ±13%.

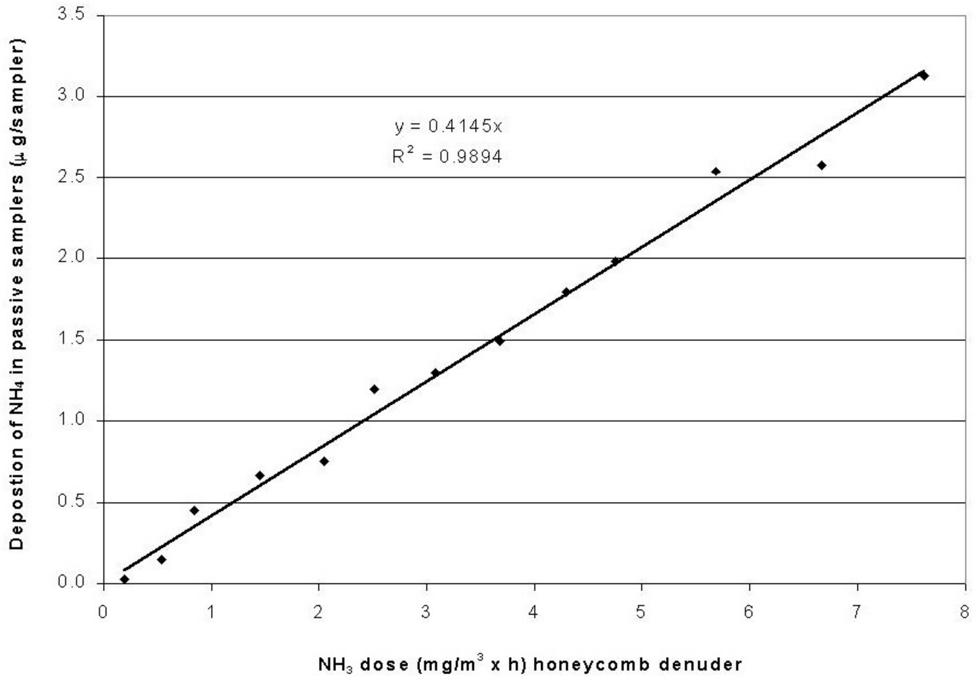

**FIGURE 2.** Comparison of ammonia passive samplers (µg of $NH_4^+$ deposited per sampler) to ammonia dose (mg $m^{-3}$ × h) measured with a honeycomb denuder system.

All samplers were placed under protective caps to avoid wind and rain. Unexposed filters for the different pollutants were kept at room temperature as references for each exposure period. At the end of each exposure period, filters were collected, placed in airtight bags and stored together with reference filters at –20ºC until analysis.

In addition to passive samplers, air temperature, relative humidity, wind speed and wind direction were monitored continuously at the sites located at 1223, 1826, and 2428 m elevations (Campbell Scientific Inc).

Sampler precision was obtained by calculating the percent difference between replicate filters at each site. A seasonal average was calculated from the 2-week-long average ozone exposures for each site to compare differences in ozone concentration along the elevation gradient. Data from sampling periods between June 15 and September 20, 2000 were used to compute summer averages. One-way ANOVA analysis was used to test for significant differences among sites in each exposure period and in total exposure period. Post-hoc comparisons were tested using the Fisher's Least Significant Difference test (LSD) calculated at the 5% level. Simple regressions were used to describe the relationship between pollutants and elevation. Pearson's correlation coefficients were calculated to examine relationship between air pollutants and local atmospheric variables. All statistical analyses were performed using Statistica v5.1 software package (StatSoft, Inc., U.S.).

## RESULTS

### Ozone

The performance of the ozone Ogawa passive samplers was very good with a precision between 0 and 6% and an average of 1.8%. No significant differences using Student's *t*-test were found between ozone data from passive samplers at Barton Flats and data from a co-located active moni-

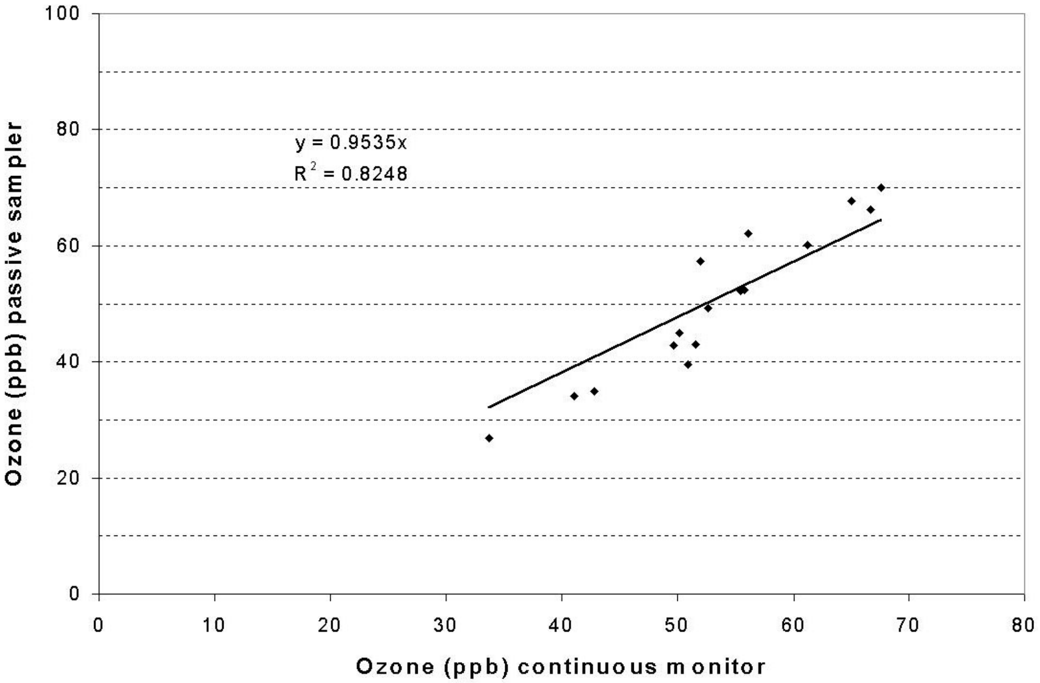

**FIGURE 3.** Comparison of ozone concentrations obtained with Ogawa passive samplers and a continuous electronic monitor.

tor (Fig. 3). Summer average 24-h $O_3$ exposure ranged from 53 ppb registered at 2111 m and at 2667 m (the highest site) to 59 ppb at the 1500 and 1826 m elevation sites (Table 1). The highest 2-week long averages were 72 to 74 ppb and the lowest value was 37 ppb (Fig. 4). Two-way ANOVA analysis showed that both elevation and time were significantly ($p < 0.001$) affecting $O_3$ concentrations but $O_3$ levels did not show a linear relationship with elevation. Considering the entire exposure period, $O_3$ levels were lower at the highest and at 2111 m elevation sites (Fig. 5). The vertical distribution of $O_3$ varied in the different exposure periods. In general and especially during the periods with higher $O_3$ levels, concentrations increased between 1223 and 1502 m, remained high at 1826 m and then decreased from that point in some periods or decreased just at the highest site in other periods. The highest $O_3$ concentrations were recorded between July 25 and August 22 and the lowest at the beginning of autumn (September 20 to October 17) and during the period August 22 to September 5.

**TABLE 1**
**Summer Means (June 15 to September 20) of Different Air Pollutants along the Elevation Gradient in the San Bernardino Mountains during Summer 2000 (Means ± SD)**

| Elevation (m) | $O_3$ (ppb) | $NO_2$ (ppb) | $HNO_3$ ($\mu g\ m^{-3}$) | $NH_3$ ($\mu g\ m^{-3}$) |
|---|---|---|---|---|
| 1223 | 55.6 ± 11.2 | 6.4 ± 1.1 | 5.1 ± 1.7 | 6 ± 1.4 |
| 1502 | 59.2 ± 8.7 | 4.9 ± 0.7 | 4.3 ± 1.4 | 5.2 ± 1.8 |
| 1826 | 58.0 ± 9.9 | 3.8 ± 0.5 | 2.4 ± 0.8 | 3.2 ± 1.3 |
| 2111 | 53.7 ± 7.3 | 2.9 ± 0.4 | 2.2 ± 0.8 | 3.9 ± 1.7 |
| 2428 | 57.2 ± 7.2 | 2.2 ± 0.4 | 2.5 ± 0.8 | 4.4 ± 1.7 |
| 2667 | 53.5 ± 6.4 | 1.5 ± 0.1 | 1.9 ± 0.5 | 2.5 ± 1.1 |

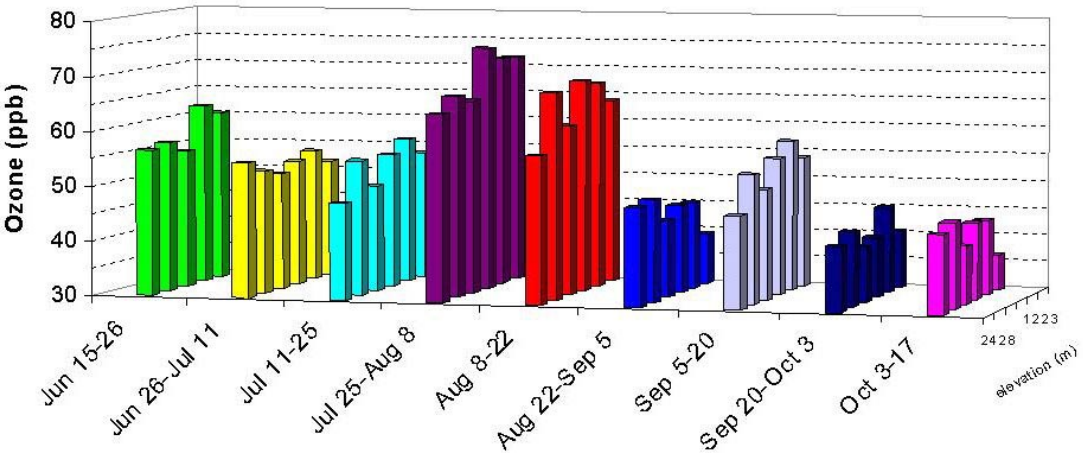

**FIGURE 4.** Concentrations of ozone (ppb) determined with passive samplers exposed during 2-week long periods during summer 2000.

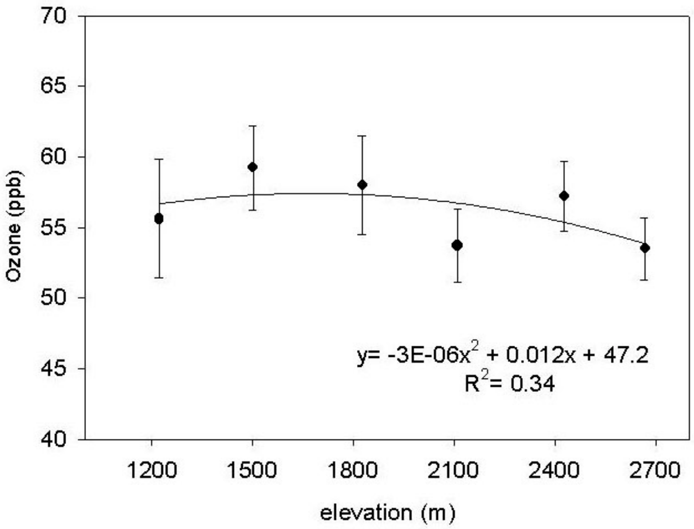

**FIGURE 5.** Changes of seasonal means of ozone (ppb) along the elevation gradient in the San Bernardino Mountains. Means ± SE.

Ozone concentrations were significantly correlated with temperature and relative humidity (Table 2). High $O_3$ concentrations were associated with high temperature and low relative humidity. When the relation between ozone levels and meteorological variables was analyzed independently for each site, the correlation with relative humidity was significant only at the lowest elevation site, where $O_3$ levels also showed significant correlation with wind speed.

## Nitrogen Oxides

Precision of Ogawa filters for $NO_2$ and $NO_x$ was slightly lower than for ozone with averages of 4 and 8%, respectively. Nitric oxide (NO) concentrations were calculated based on the difference between nitrite produced in the $NO_x$ and $NO_2$ samplers. Nitric oxide concentrations at

**TABLE 2**
**Correlation Coefficients between Meteorological Variables and Air Pollutants (n = 25–26)***

| | $O_3$ (ppb) | $NO_2$ (ppb) | $HNO_3$ (µg $m^{-3}$) | $NH_3$ (µg $m^{-3}$) |
|---|---|---|---|---|
| Temperature (°C) | 0.627<br>p<0.005 | 0.347<br>n.s. | 0.785<br>p<0.001 | 0.431<br>p<0.05 |
| Relative humidity (%) | -0.63<br>p<0.005 | 0.128<br>n.s. | -0.684<br>p<0.001 | -0.222<br>n.s. |
| Wind speed (m $s^{-1}$) | 0.018<br>n.s. | -0.238<br>n.s. | 0.182<br>n.s. | 0.296<br>n.s. |

*Data from sites at 1123, 1826, and 2428 m elevation.

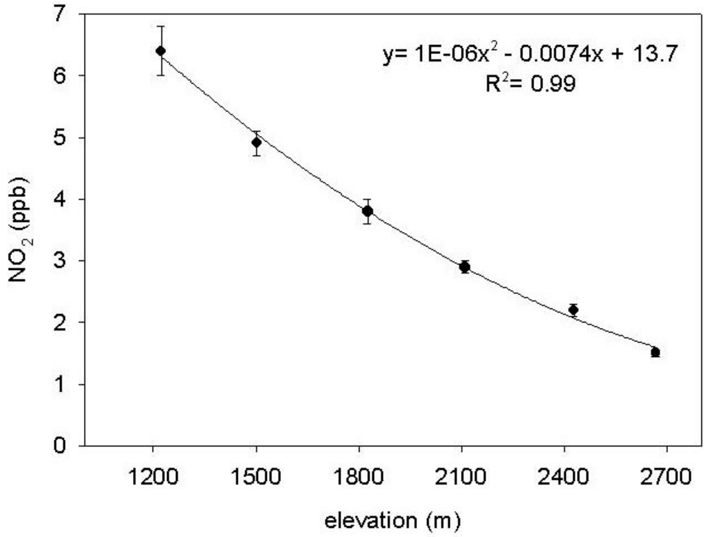

**FIGURE 6.** Changes of seasonal means of $NO_2$ (ppb) along the elevation gradient in the San Bernardino Mountains. Means ± SE.

all the sites along the gradient were around 1 to 2 ppb that is within the measurement uncertainty of these devices.

Significant effects of elevation on nitrogen dioxide levels were detected ($p < 0.001$) showing a strong negative correlation with decreasing concentrations with increasing elevation ($R^2 = 0.99$; Fig. 6). Summer averages of $NO_2$ ranged from 1.5 ppb to 6.4 ppb (Table 1). Similarly, time also induced significant variations ($p < 0.001$) that were less pronounced as the elevation increased. The highest $NO_2$ values occurred during the last exposure period (October 3 to17) and during the period August 22 to September 5 (Fig. 7). Two-week period $NO_2$ averages were between 1 and 9.7 ppb. Temperature, relative humidity, and wind speed were significantly correlated with $NO_2$ concentrations only at the lowest elevation site. High $NO_2$ values were associated with low temperatures, low wind speeds, and high relative humidity.

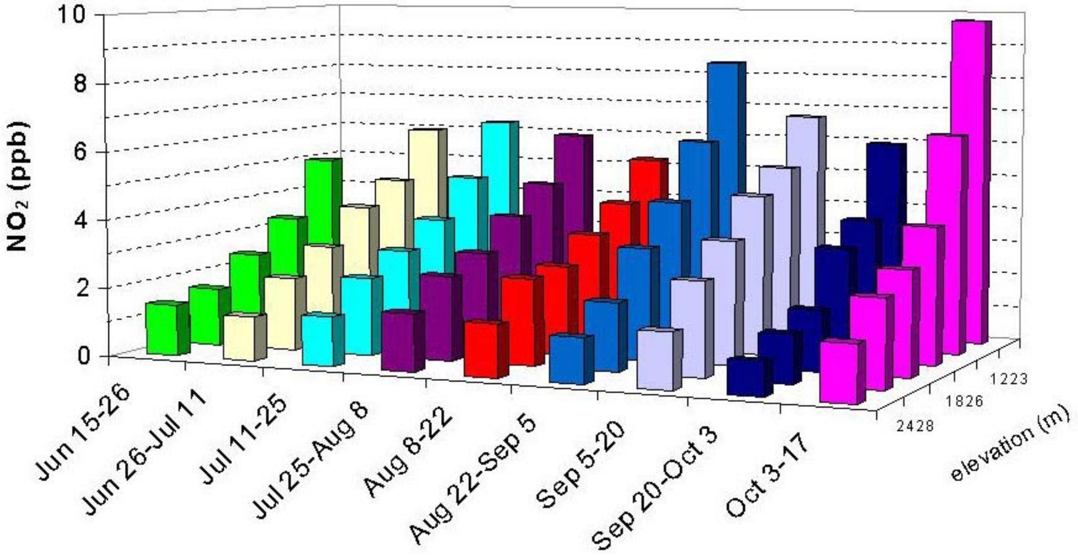

**FIGURE 7.** Concentrations of $NO_2$ (ppb) determined with passive samplers exposed during 2-week long periods during summer 2000.

## Nitric Acid Vapor

Average precision of $HNO_3$ samplers was 11% among co-located filters. Both elevation and time had significant effects on $HNO_3$ exposure ($p < 0.001$). Summer average $HNO_3$ concentrations were negatively correlated with elevation ($R^2 = 0.93$, Fig. 8) ranging from 5.1 µg $m^{-3}$ at the lowest site to 1.9 µg $m^{-3}$ at the highest site (Table 1). The two sites located at the lower elevations had consistently higher $HNO_3$ concentrations than the rest of the sites up on the gradient. All the sites had the highest $HNO_3$ concentrations in the middle of the season (from July 11 to August 22) and the lowest values at the beginning of autumn and during the period August 22 to September 5 (Fig. 9). Two-week averages ranged between 6.5 µg $m^{-3}$ registered at the lowest site of the gradient during July and August and 0.66 µg $m^{-3}$ recorded in October at 2111 m of elevation.

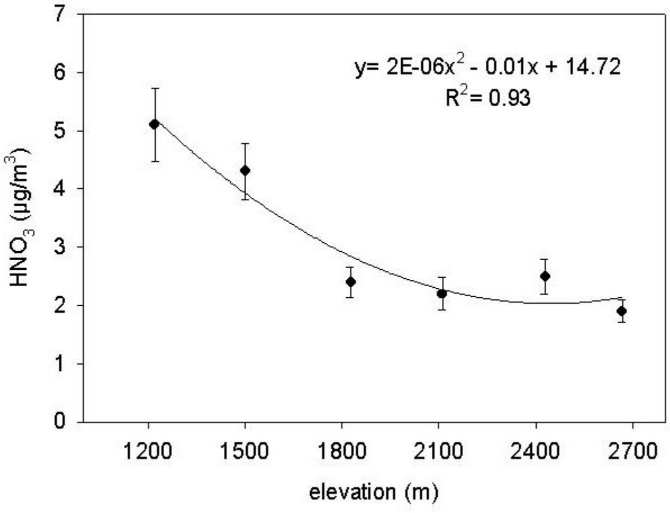

**FIGURE 8.** Changes of seasonal means of $HNO_3$ (µg $m^{-3}$) along the elevation gradient in the San Bernardino Mountains. Means ± SE.

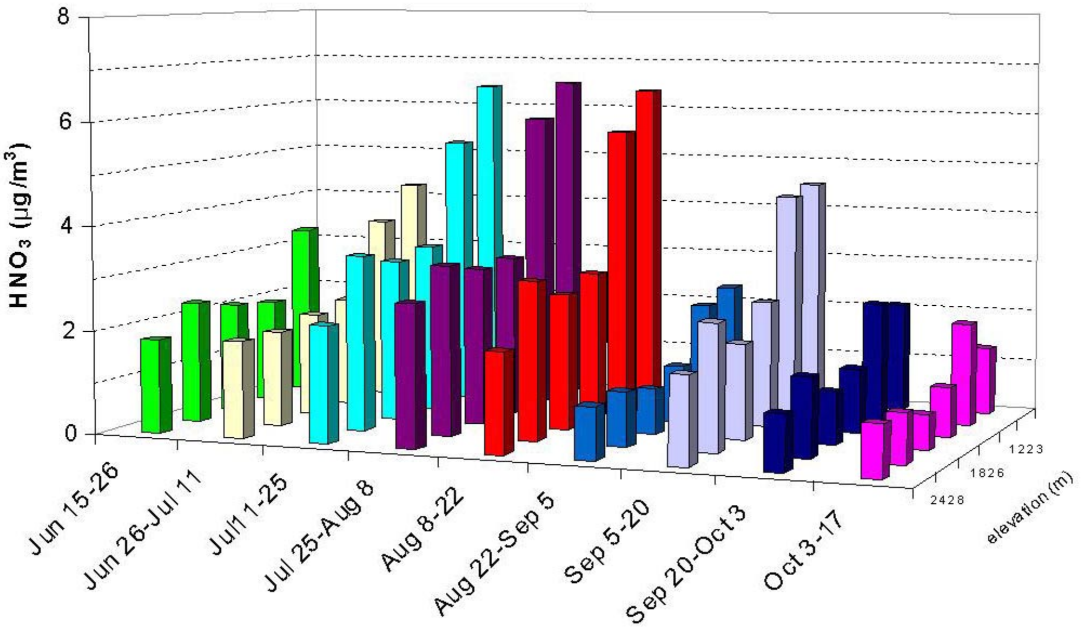

**FIGURE 9.** Concentrations of $HNO_3$ (µg $m^{-3}$) determined with passive samplers exposed during 2-week long periods during summer 2000.

Similarly to $O_3$, $HNO_3$ vapor exposure was significantly correlated with temperature and relative humidity (Table 2), relationships that were observed in all the sites. In the lowest site (1223 m), $HNO_3$ was also correlated to wind speed.

## Ammonia

Average precision of diffusion tubes for measuring $NH_3$ was 23% with most of the values ranging from 5 to 35%. The highest summer average of $NH_3$ concentrations was recorded at the lowest elevation site (6 µg $m^{-3}$) while the lowest average at the highest elevation (2.5 µg $m^{-3}$; Table 1). Although elevation caused significant changes in $NH_3$ concentrations ($p < 0.001$), $NH_3$ levels did not consistently decrease with increasing elevation (Fig. 10). During most of the exposure periods, $NH_3$ concentrations showed minimum values at sites located at 1826 m and 2667 m (highest site) and maximum values at the two lower sites (1223 and 1502 m) and at 2428 m (Fig. 11). This vertical distribution was different at the end of summer and beginning of autumn when the lower sites presented higher $NH_3$ levels than the other sites up on the gradient. A significant effect of time ($p < 0.001$) was also detected with the highest values recorded in the June 15–26 period and in the middle of the season (July 22 through August 22). Although ammonia concentrations seemed to show correlations with temperature and wind direction, these meteorological variables were not significantly correlated with $NH_3$ concentrations when data were analyzed independently for each site.

## DISCUSSION

The particular geographical location and climatic conditions of the highly populated Los Angeles Basin are the reasons why this urban region has one of the highest air pollution exposures in the

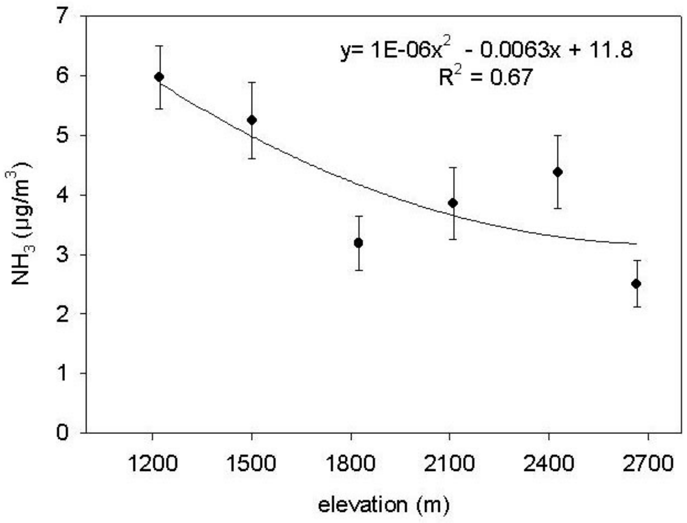

**FIGURE 10.** Changes of seasonal means of $NH_3$ (µg $m^{-3}$) along the elevation gradient in the San Bernardino Mountains. Means ± SE.

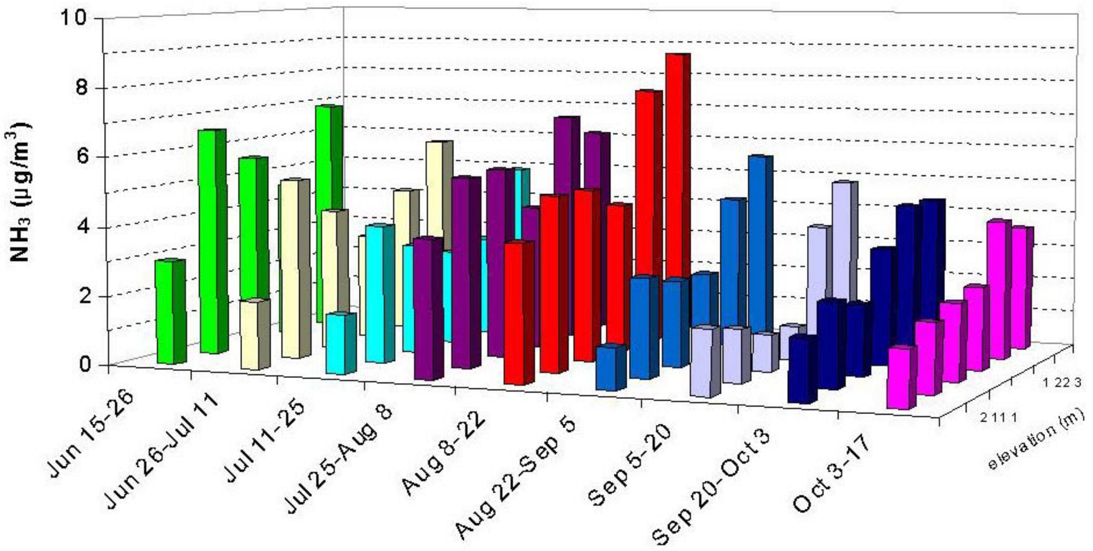

**FIGURE 11.** Concentrations of $NH_3$ (µg $m^{-3}$) determined with passive samplers exposed during 2-week long periods during summer 2000.

world. The area is surrounded by the Pacific Ocean and by several mountain ranges giving a set of meteorological conditions (high temperatures and radiation, frequent episodes of thermal inversions) which promote the formation of photochemical pollution[24]. Sea breezes cause air to flow from the ocean inland during the early morning hours and back to the ocean during the afternoon and evening. The back and forth flow of the air is constrained by the surrounding mountains which allows the air to become highly enriched with pollutants.

Summer averages of $O_3$ concentrations during the summer 2000 (53–59 ppb) were in the same range as values recorded in the summer 1995 in the same area[25]. These values were lower than summer means registered at Barton Flats (a nearby site at 2000 m of elevation) recorded during 1974–76[16] or in early 1990s[26]. These results confirm the trend towards decreasing $O_3$ concentrations that has been observed in this area in recent years[5]. Although ambient $O_3$ levels in southern California are decreasing, concentrations are still much higher than the 30 to 45 ppb considered as a background in northern hemisphere[27]. Phytotoxic effects on plants at the recorded levels have already been described in these mountains[6].

Nitric oxide (NO) concentrations were very low during all the summer and $NO_2$ levels were between 1.5 and 6.4 ppb depending on the elevation. NO is the compound preferentially emitted from fossil fuel combustion but it readily reacts with $O_3$ or peroxyl radicals to form $NO_2$. Oxidation processes that occur during photochemical smog formation result in NO concentrations in forest areas that are generally low. Concentrations of NO and $NO_2$ found in the San Bernardino Mountains are typical for other forest locations in California mountains[20,28,29].

Concentrations of $HNO_3$ vapor (summer averages of 1.9 to 5.1 µg $m^{-3}$ depending on elevation) were similar to those found at other locations in southern California, western Sierra Nevada and Europe[13,20,26,30]. These levels are lower than concentrations found at Tanbark Flat in the San Gabriel Mountains (east of San Bernardino Mountains and closer to Los Angeles[31]), but are an order of magnitude higher than 0.18 to 0.36 µg $m^{-3}$ of $HNO_3$ recorded at the North American remote locations or in the less polluted area of eastern Sierra Nevada[13]. The measured $HNO_3$ vapor concentrations recorded during the summer 2000 maintained the levels recorded in Barton Flats during the early 1990s[30].

Ammonia concentrations at higher elevation sites (summer average 2.5 to 4.4 µg $m^{-3}$), although close to the detection limit for this method, were in the same range as those recorded at other mountain forests in California[13,20,26,30]. Summer average of $NH_3$ recorded at the lowest elevation site (6 µg $m^{-3}$ at 1223 m of elevation) was high, indicating the proximity of this site to pollutant source locations However, this value was lower than $NH_3$ levels recorded at locations with more agricultural activities[32,33]. Ammonia levels during summer 2000 were higher than concentrations registered at Barton Flats in the early 1990s[13,26,30]. Summer concentrations of $NH_3$ along the elevation gradient were slightly higher than $HNO_3$ vapor, reflecting a strong influence of agricultural emissions from the western part of the Riverside county on air quality in the San Bernardino Mountains.

The highest concentrations of ozone and $HNO_3$ vapor occurred during mid-summer. Ammonia concentrations were also high at that time but similar levels were recorded at the beginning of the summer. By contrast $NO_2$ levels increased at the end of summer. Similar seasonal behavior of $NH_3$, $HNO_3$, and $O_3$ has been described in the western Sierra Nevada[29,34]. The high correlation found between $O_3$ and $HNO_3$ ($r = 0.648$, $p < 0.001$) indicates the photochemical origin of $HNO_3$ vapor. Such a co-occurrence of air pollutants has been described in areas affected by photochemical smog[35]. Photochemical origin of these pollutants is also indicated by the correlation between higher levels of ozone and nitric acid vapor and high temperatures and low relative humidity as found by other authors[34,36,37]. The relationships between concentrations of air pollutants and meteorological variables became weaker with increasing elevation. These low correlation coefficients suggest that integration measurements obtained with passive samplers not only depend on the formation pathways, but also on deposition and removal mechanisms.

Distribution of ozone has been extensively studied since it is considered to be one of the most phytotoxic air pollutants affecting a wide range of ecosystems. Ozone concentrations depend on site-specific characteristics such as the influence of regional meteorology, topographical features and atmospheric conditions that affect local photochemistry and transport processes of pollutants from the source regions. No conclusive statement can be made concerning relationship between $O_3$ exposure and elevation. Several authors have found increases of ozone exposure with elevation in central Europe[38,39,40]. Similarly, Brace and Peterson[41] described a positive correlation between $O_3$ and elevation in the Cascade Mountains (Washington, U.S.) in three of the four transects analyzed recording $O_3$ levels up to 2100 m of elevation. This pattern has also been found in some mountain ranges in eastern U.S.[34]. However, other elevation gradients monitored in the eastern U.S.[42] and in the San Gabriel Mountains near Los Angeles urban area[43] have shown no ozone increases with elevation. Lefohn[44] introduced caution in generalizing about the relationship between elevation and $O_3$ exposure and emphasized the value of comparing data for multiple years on elevation gradients.

In the San Bernardino Mountains, we found that the vertical distribution of $O_3$ during the summer of 2000 varied in different exposure periods. This variability was also detected in a similar study performed in 1991 using a network of passive samplers along the north and south slopes of the same mountain[43]. In the periods with higher ozone levels, $O_3$ concentrations increased with elevation up to about 1500 m and then gradually decreased with elevation. A previous study performed in this same area during the late summer of 1995 pointed out that the highest $O_3$ exposure was at 1585 m on an elevation gradient from 1192 to 2800 m[25]. Above 1585 m, $O_3$ concentration decreased with elevation in two of the three periods considered. Miller et al.[16] also found sharp increases of $O_3$ levels between valleys (400 m of elevation) compared to mountain locations (around 1800 m) in other locations in the San Bernardino Mountains. This convex shaped curve with maximum $O_3$ values in the height range of 1000 to 2000 m has been described in some location in the Alps[45]. Similarly, a study performed in the western slope of Sierra Nevada (Sequoia National Park, California, U.S.) during the summer 1999 also showed increasing $O_3$ levels from 500 to 1200 m of elevation and then concentrations decreased with increasing elevation up to 2200 m[20]. However, in that case pollutant dilution with distance was probably more important than increasing photochemical processes with elevation. Stockwell et al.[27] also found the highest $O_3$ levels at the altitude of about 1000 m but this behavior depended on atmospheric conditions, especially solar radiation. Moreover, some authors have pointed out that the relationship between $O_3$ levels and elevation might depend on daytime. During the night, sites located below the nocturnal boundary layer usually experience a decrease in $O_3$ concentrations caused by chemical consumption and deposition of $O_3$, while those sites above the boundary layer do not experience such a depletion of $O_3$ due to the absence of mixing[34,38]. In such conditions higher $O_3$ levels are found at higher elevation sites. But during the day, high temperatures along with wind patterns increase the vertical mixing, and thus differences of $O_3$ concentrations depending on elevation decrease. Because passive samplers integrate exposure over several days periods, these variations of $O_3$ with elevation on a short time scale cannot be assessed. All these results suggest that the vertical distribution of $O_3$ concentrations vary with terrain features of individual mountain ranges and also with climate.

Distribution of N compounds has received much less attention than ozone. Concentrations of nitrogenous pollutants decreased with increasing elevation along the elevation gradient surveyed in the San Bernardino Mountains, although patterns were slightly different for $NO_2$, $NH_3$, and $HNO_3$ vapor. While $NO_2$ decreased consistently with elevation, $NH_3$ and $HNO_3$ showed a sharp decrease between 1200 to about 1800 m of elevation but concentrations remain unchanged above that elevation. A decrease of $HNO_3$ and $NH_3$ with elevation and distance from pollutant source areas has been described in Sequoia National Park along a gradient from 500 to 2200 m[20], although in the same study $NO_2$ concentrations did not show a clear pattern of variation with elevation. Aneja et al.[34] also found decreasing $HNO_3$ concentrations with increasing elevation in Mt. Mitchell in North Carolina. The variability of $NH_3$ and $HNO_3$ concentrations above certain elevation could be related to particular characteristics of exposure or deposition regimes at different sites.

Concentrations of N pollutants in the San Bernardino Mountains during the summer 2000 were elevated compared to remote locations, although no direct damage to vegetation can be expected at the recorded levels[46,47]. However, these nitrogen concentrations might be providing substantial amounts of N to forest ecosystems causing major perturbations in biogeochemical cycling of nitrogen. Nitrogen deposition is believed to stimulate forest productivity in areas receiving moderate N deposition but N inputs may also have a negative effect on tree growth depending on forest soils[48]. Symptoms of nitrogen saturation have already been described in mixed-conifer forests in the western end of San Bernardino Mountains[6,49]. Similarly, recent changes observed in species composition of coastal sage scrub stands near Los Angeles have been associated with significant changes in nitrogen fertility caused by the deposition of N pollutants[50,51,52]. The long-term ecological effects of this nitrogen excess on forest ecosystems are still unknown. Atmospheric N deposition combined with $O_3$ exposure caused shifts in biomass allocation in ponderosa pines growing in the San Bernardino Mountains decreasing root biomass, root carbohydrate concentration and foliar retention[11,12,53]. Reduction of root biomass may impair the ability of trees to withstand other environmental stress and might be considered as one of the first steps in forest health degradation induced by air pollution. The progression toward a deciduous growth pattern, higher shoot:root ratios, increasing depths of litter and elevated nitrate concentrations in soil may induce species replacement with nitrophilous and ozone-tolerant tree species[10,54]. Since it seems likely that projected increases in road traffic will keep concentrations of air pollutants increasing, the San Gorgonio Wilderness area ecosystems may be even more affected by air pollution in the future.

## CONCLUSIONS

Concentrations of ozone and nitrogen pollutants in the San Bernardino Mountains during summer 2000 were elevated. Although N pollutants levels were not high enough to directly threaten vegetation, N deposition might be affecting forest ecosystems by altering biogeochemical cycling of nitrogen, as it has been already observed in other areas of the San Bernardino Mountains. No clear pattern of vertical distribution of ozone was detected in this area suggesting that distribution of ozone concentrations depends on topographic features and climatic conditions. Nitrogenous pollutants ($NO_2$, $HNO_3$, $NH_3$) decreased with increasing elevation. Since interactive effects of different air pollutants have been detected on forest trees, characterization of the distribution of co-occurring pollutants should be addressed to provide information needed for air pollution risk assessment on forest ecosystems.

## ACKNOWLEDGEMENTS

This research was supported by USDA Forest Service PSW Research Station and Sierra Nevada Monitoring Program. The authors thank David Jones, Anthony Gomez, Ronald Johnson and Pamela Padgett of the USDA Forest Service PSW Research Station for their valuable help in field work; Diane Alexander, Antonio Davila and Enrique Jimenez for chemical analyses; Susan Schilling for preparing Fig. 1. The Spanish Ministry of Education and Culture and the Fulbright Commission provided financial support to Rocío Alonso.
